# Draft Genome Sequence of *Micromonospora* sp. Strain MW-13, a Bacterial Strain with Antibacterial Properties and Plant Growth Promotion Potential Isolated from the Rhizosphere of Wheat in Iran

**DOI:** 10.1128/MRA.01375-18

**Published:** 2019-01-10

**Authors:** Ghazaleh Jahanshah, Henrike Miess, Tobias Busche, Christian Rückert, Jörn Kalinowski, Hassan Rokni-Zadeh, Harald Gross

**Affiliations:** a Department of Pharmaceutical Biology, Institute of Pharmaceutical Sciences, University of Tübingen, Tübingen, Germany; b German Centre for Infection Research (DZIF), Partner Site Tübingen, Tübingen, Germany; c Technology Platform Genomics, CeBiTec, Bielefeld University, Bielefeld, Germany; d Department of Medical Biotechnology and Nanotechnology, Zanjan University of Medical Sciences, Zanjan, Iran; University of Rochester School of Medicine and Dentistry

## Abstract

Strain MW13 exhibited broad-spectrum antibacterial activity toward Gram-positive and Gram-negative pathogens. The 7.1-Mb draft genome gives insight into the complete secondary metabolite production capacity and reveals genes putatively responsible for its antibacterial activity, as well as genes which contribute to plant growth promotion.

## ANNOUNCEMENT

As part of our ongoing efforts to investigate natural products from rhizosphere bacteria, which are relevant in an agricultural or pharmaceutical context (1–6), we recently isolated and characterized 80 rhizospheric soil samples from wheat, barley, and clover fields, as well as turf grass and buckthorn trees in the Isfahan province in central Iran. In a subsequent screening panel, isolate MW13 exhibited antibacterial activity. Based on 16S rRNA gene sequence similarity, strain MW13 was identified as a Micromonospora sp. The most closely related type strains to MW13 are Micromonospora echinofusca DSM 43913 and Micromonospora auratinigra DSM 44815, both with 99% sequence identity. Bacteria of the genus Micromonospora have long been recognized as an important source of aminoglycoside antibiotics (7) but also recently for their potential regarding biocontrol and breakdown of cellulosic biomass for biofuels (8). They play an important role in soil ecology, biodegradation, and plant growth promotion, but little is known about how these microbes accomplish these numerous functions. Therefore, we aimed to determine the whole-genome sequence of strain MW13 to reveal the genetic background of its antibacterial capacity, as well as to provide a resource to study factors involved in plant association and potential biocontrol properties.

Strain MW13 was isolated from the rhizosphere of Triticum aestivum L. Five grams of roots plus adhering rhizosphere soil were suspended in 50 ml of sterile phosphate-buffered saline (9) in 100-ml Erlenmeyer flasks and shaken for 30 min at 30°C. The suspension was filtered, and a dilution series was prepared. The filtered suspensions were plated onto King’s B agar (10). After 4 days at 30°C, strain MW13 along with 10 further bacterial colonies could be distinguished and separately isolated based on their morphological appearance.

Strain MW13 was grown in 15 ml Trypticase soy broth (TSB) overnight at 30°C on a rotary shaker (180 rpm). For genomic DNA (gDNA) isolation, the Qiagen genomic DNA purification kit was used in combination with 100/G Genomic-tips, according to the manufacturer’s protocol, except that for the bacterial lysis, the handled volumes were doubled, and the incubation time at 50°C was prolonged until a clear lysate was obtained.

A paired-end library was constructed using the TruSeq PCR-free kit (Illumina), according to the manufacturer’s protocol, and subjected to sequencing using an Illumina HiSeq 1500 platform in a 2 × 250-bp run. A total of 2,602,577 paired-end reads were obtained, and the data were quality checked using FastQC version 0.11.5, operating with default parameters. With an average quality score of Q37 for the forward reads and Q34 for the reverse reads, the reads were trimmed from the 3′ end, removing bases with a score below Q20. The *de novo* assembly was performed utilizing Newbler version 2.8, with default parameters, using a subset of 2.6 million reads and screening against the phiX sequence as vector contamination. Overall, 2,597,997 reads were assembled into a 7,086,037-nucleotide draft at 84.7-fold coverage. The resulting draft genome sequence consists of 152 contigs in total (average contig size, 57,095 bp) in 41 scaffolds, with a G+C content of 73.3%. The assembled contigs were annotated with the PROKKA version 1.11 pipeline (11), resulting in the annotation of 6,184 coding sequences.

Automated secondary metabolism analysis using antiSMASH 4.0.2 (12) predicted 25 biosynthesis gene clusters (BGCs). Eight of these matched known clusters for the biosynthesis of desferrioxamine B (13, 14), sioxanthin (15), and landomycin (16). The remaining clusters were predicted to encode 2 terpenoid-, 4 nonribosomal peptide synthetase (NRPS)-, 9 polyketide synthase (PKS)-, 1 hybrid-NRPS-PKS-, and 6 ribosomally synthesized and posttranslationally modified peptide (RiPP)-based compounds.

### Data availability.

This whole-genome sequence (WGS) project and the 16S rRNA gene sequence have been deposited at DDBJ/ENA/GenBank under the accession numbers QKKX00000000 and MK045809, respectively. The raw sequencing data are available from the Sequence Read Archive (SRA) under the accession number SRR7949780.
